# Passive acoustic monitoring reveals group ranging and territory use: a case study of wild chimpanzees (*Pan troglodytes*)

**DOI:** 10.1186/s12983-016-0167-8

**Published:** 2016-08-08

**Authors:** Ammie K. Kalan, Alex K. Piel, Roger Mundry, Roman M. Wittig, Christophe Boesch, Hjalmar S. Kühl

**Affiliations:** 1 Department of Primatology, Max Planck Institute for Evolutionary Anthropology, Deutscher Platz 6, 04103 Leipzig, Germany; 2 School of Natural Sciences and Psychology, Liverpool John Moores University, James Parsons Building, Rm 653, Byrom Street, Liverpool, L3 3AF UK; 3 Ugalla Primate Project, Kigoma, Tanzania; 4 Department of Developmental and Comparative Psychology, Max Planck Institute for Evolutionary Anthropology, Deutscher Platz 6, 04103 Leipzig, Germany; 5 Taï Chimpanzee Project, Centre Suisse de Recherches Scientifiques, BP 1301, Abidjan 1, Côte d’Ivoire; 6 Wild Chimpanzee Foundation, Deutscher Platz 6, 04103 Leipzig, Germany; 7 German Centre for Integrative Biodiversity Research (iDiv) Halle-Jena-Leipzig, Deutscher Platz 5e, 04103 Leipzig, Germany

**Keywords:** Animal communication, Autonomous recording unit, Bioacoustics, Buttress drumming, Loud calls, Ranging pattern

## Abstract

**Background:**

Assessing the range and territories of wild mammals traditionally requires years of data collection and often involves directly following individuals or using tracking devices. Indirect and non-invasive methods of monitoring wildlife have therefore emerged as attractive alternatives due to their ability to collect data at large spatiotemporal scales using standardized remote sensing technologies. Here, we investigate the use of two novel passive acoustic monitoring (PAM) systems used to capture long-distance sounds produced by the same species, wild chimpanzees (*Pan troglodytes*), living in two different habitats: forest (Taï, Côte d’Ivoire) and savanna-woodland (Issa valley, Tanzania).

**Results:**

Using data collected independently at two field sites, we show that detections of chimpanzee sounds on autonomous recording devices were predicted by direct and indirect indices of chimpanzee presence. At Taï, the number of chimpanzee buttress drums detected on recording devices was positively influenced by the number of hours chimpanzees were seen ranging within a 1 km radius of a device. We observed a similar but weaker relationship within a 500 m radius. At Issa, the number of indirect chimpanzee observations positively predicted detections of chimpanzee loud calls on a recording device within a 500 m but not a 1 km radius. Moreover, using just seven months of PAM data, we could locate two known chimpanzee communities in Taï and observed monthly spatial variation in the center of activity for each group.

**Conclusions:**

Our work shows PAM is a promising new tool for gathering information about the ranging behavior and habitat use of chimpanzees and can be easily adopted for other large territorial mammals, provided they produce long-distance acoustic signals that can be captured by autonomous recording devices (e.g., lions and wolves). With this study we hope to promote more interdisciplinary research in PAM to help overcome its challenges, particularly in data processing, to improve its wider application.

**Electronic supplementary material:**

The online version of this article (doi:10.1186/s12983-016-0167-8) contains supplementary material, which is available to authorized users.

## Background

Any study of animal behavioral ecology requires basic knowledge about home range and habitat use within the natural environment to grasp the fundamental social and ecological selection pressures acting on individual fitness. Researchers have known for some time that determining the home range or territory of wild animals requires a long-term investment of data collection [1, 2]. This is made more difficult for large-ranging mammals, where following individuals is physically demanding or impossible (e.g., cetaceans, bats), and invasive options such as tracking devices or radio collars are expensive and may pose a risk to wild animals [3]. Hence, alternative cost-effective methods to non-invasively monitor and track wildlife are needed.

At present, indirect indices of animal presence, such as feces, tracks, and nests, can provide us with evidence of ranging and grouping behavior. For example, genotypes can be extracted from dung and hair surveys that, via sample association patterns, can provide data on group structure and composition [4, 5]. Specific to great apes, sleeping nest sites provide information on the size of a group, whilst their spatial distribution and clustering can be used to infer territories [6]. Nonetheless, indirect monitoring methods still require years of longitudinal data collection to ensure an adequate sampling effort has been achieved to reflect the true size of a group or territory [1]. Alternatively, direct observations, where individuals are followed by researchers to collect data on ranging and behaviour, can help to minimize sampling bias which is of concern when studies rely on indirect sampling methods [3]. However, some wild animals live in environments (e.g. underwater) or are active at times (e.g. night) inherently difficult for researchers to visually monitor, whilst others have learned that people are dangerous and actively avoid them.

To overcome such challenges, marine biologists have long applied passive acoustic monitoring (PAM), a non-invasive method of monitoring wildlife using sound recording devices [7, 8]. The application of PAM is made even more feasible in an aquatic environment because calls can propagate much further in water than on land [9]. Through the use of sonar and hydrophone arrays, researchers have been monitoring the movements of cetaceans for decades by tracking their natural acoustic behavior [8]. Similarly, the use of remote audio recordings to monitor birds [10], bats [11] and insects [12] has also proven effective, facilitated by the high stereotypy of the sounds produced by these animals.

More recently, researchers working in terrestrial environments which limit visual observations, such as in dense rainforests or with cryptic animals, have begun using PAM. It has been successfully employed for the study of tropical birds [13, 14] and land mammals such as forest elephants [15, 16], Bornean orangutans [17], as well as multi-species systems [18, 19], but one limiting factor in all terrestrial applications continues to be effective automated approaches for mining the PAM data to extract calls of interest. The high variability of many mammalian vocalizations, and complex background noise present in terrestrial ecosystems [20] have hindered the progress of PAM for land mammals, including our closest living relatives, the great apes. Generally speaking, research and conservation of great apes has garnered great interest due to their genetic and behavioral similarities to humans [21]. Yet, much of what we know about great apes comes from a few groups that have been habituated by researchers to tolerate the presence of human observers; however, this requires an investment of years and is neither feasible nor ethical for all wild populations [22, 23]. As such, non-invasive monitoring methods, such as PAM, are becoming increasingly useful to coordinate conservation efforts for remaining healthy populations and to produce results in a cost-effective and efficient way. Therefore, we investigated the potential of PAM to provide information about the ranging behavior of wild chimpanzees (*Pan troglodytes*), a territorial and vocally conspicuous mammal.

Forest-dwelling chimpanzees are known to have territories ranging from 7 km^2^ (Sonso, Uganda [24]) to 31 km^2^ (East group, Taï, Côte d’Ivoire [6]). While we know less about savanna-woodland dwelling chimpanzees, estimates of their territories range from 72 km^2^ (Semliki, Uganda [25]) to 239 km^2^ (Assirik, Senegal [26]). Individuals of a single group, or chimpanzee ‘community’, spend the majority of their time in the core of their home range, usually representing about 75–90 % of their total territory [27, 28]. Chimpanzees are also xenophobic, exhibiting sometimes fatal aggression during inter-community encounters [29]. Accordingly, chimpanzees are observed to modify their behavior in the periphery of their territory as a response to an elevated degree of risk [28, 30]. Here, chimpanzees engage in territorial boundary patrols during which mostly adult males of the community coordinate their behavior to remain silent and vigilant whilst inspecting the periphery of their territory for possible excursions by stranger chimpanzees [29, 31]. Because chimpanzees exhibit these changes in their vocal behavior when in the periphery of their territory, we wanted to test whether by remotely monitoring their vocalizations throughout their territory we could infer their ranging patterns. A previous PAM study has already shown that primate calls can be used to obtain reliable estimates of occurrence for chimpanzees and sympatric forest monkeys [32] which, if monitored overtime, could be used to assess population trends.

In this study, we investigated whether remote acoustic monitoring of chimpanzees, living in both tropical forest and in savanna-woodland habitat, could be used to obtain information about a group’s ranging behavior and thereby be used as a tool to aid monitoring wild populations. We focused on long-distance sounds produced by chimpanzees, namely ‘drumming’ with hands and feet on the buttress roots of trees and the ‘pant hoot’ vocalization [33, 34]. The pant hoot is a long, compound call composed primarily of ‘hoos’ and ‘screams’ [35]. Both are long range acoustic signals that can be heard at a distance of up to 1 km [35, 36]. These calls are particularly significant for chimpanzees since they live in a fission-fusion society in which community members travel in smaller sub-groups, or parties. The long-distance pant hoots and drums function to maintain contact with individuals travelling in different parties and to coordinate group movement [33, 37]. First, we tested whether detections of chimpanzee drums or pant hoots on autonomous acoustic recording devices were predicted by known chimpanzee activity. Second, if these long distance acoustic signals proved to be reliable indicators of chimpanzee activity, we further examined whether boundaries of chimpanzee communities could be identified spatially using PAM, based on the reasoning that chimpanzee activity is highest in the core area of the territory [27]; therefore, vocalizations were expected to be more frequent in the core [28]. Third, we asked whether PAM could be used to observe spatiotemporal changes in territory use given that chimpanzees are expected to exploit different areas of their home range based on fluctuations in resource availability [38, 39]. Finally, by testing the application of this method for the same species but in two vastly different habitats we aimed to determine the degree of usefulness of PAM for other populations of chimpanzees as well as other mammals with similar behavioral ecology.

## Methods

### Data collection

#### PAM data collection

In the Taï National Park, Côte d’Ivoire, we collected audio recordings using 20 autonomous recording units (ARUs: Songmeter SM2+ from Wildlife Acoustics) placed in a systematic grid covering 45 km^2^ of primary evergreen forest (Table 1). We mounted ARUs on small trees 1–2 m from the ground since drums and pant hoots are primarily produced by chimpanzees while traveling on the ground, and therefore are expected to propagate close to the ground. The ARUs were placed within the research area of the Taï Chimpanzee Project, spanning two habituated, neighboring chimpanzee communities: South and East groups [6, 27]. At the time of the study the South group comprised 19 chimpanzees plus five dependent offspring, and the East group totaled 23 individuals plus seven dependent offspring. A previous study has estimated the South group home range to be 27 km^2^ and 31 km^2^ for the East group [6]. ARUs had a maximum detection distance of 1 km for chimpanzee sounds [32]. ARUs recorded in stereo at 16 kHz sampling frequency and an amplitude resolution of 16 bits/s. The devices were pre-programmed to record for 30 min on the hour, from 6.00 am to 17.30 pm (6 h/day). Periodically, some ARUs did not work due to technical problems, thus a total of 12,851 h of ARU recordings were collected across 137 days from November 2011 to May 2012. These audio data are available on request from the IUCN/SSC A.P.E.S database (http://apesportal.eva.mpg.de/).

**Table 1:** Overview of data collected using autonomous recording devices at two chimpanzee field sites

# of recording months	habitat	chimpanzee population	chimpanzee density (inds/ km^2^)	# of devices	study area	acoustic signal
Issa valley, Tanzania						
11	savanna-woodland	unhabituated	0.25	10	12 km^2^	pant hoot
Taï, Côte d’Ivoire						
7	rainforest	habituated	0.97	20	45 km^2^	drum

In the Issa valley, Tanzania, ten custom built solar powered acoustic transmission units (SPATUs) were deployed (Table 1) where the Ugalla Primate Project, a long-term research project, has been consistently running since 2008 [40]. The vegetation at Issa is predominantly miombo-woodland with interspersed grasslands, swamp, and riverine closed-canopy forest. The valleys are generally forested while slopes and plateaus are primarily woodlands. As such, the Issa valley represents one of the most open and driest habitats in which chimpanzees live [36, 41], and this population is currently undergoing habituation to researcher presence. Via indirect genetic sampling, it has been estimated that 67 chimpanzees comprise a single community at Issa [42]. SPATUs recorded 24 h/day, with 5.5 kHz sampling rate and an amplitude resolution of 16 bits/s using a single channel [36]. The ten SPATUs used in this study also had an effective 1 km detection distance for chimpanzee pant hoots and covered a total area of 12 km^2^ [36]. The units were installed 4–6 m above the ground in trees to access enough solar energy, and recorded for 247 days from April 2009 to February 2010. For comparability, we only included Issa recordings made during the same hours of the day as the Taï dataset, for a total of 29,640 h of SPATU data.

#### Detecting chimpanzee call events

A customized algorithm for the automated detection and classification of chimpanzee sounds was used to extract chimpanzee drums from continuous ARU recordings made in the Taï forest [19]. Only chimpanzee drums that were subsequently verified to be true positive detections were included in the final dataset, however; we also included any detections that had been misclassified as chimpanzee drum but were actually long range chimpanzee vocalizations (screams or pant hoots). The maximum recall for chimpanzee drum sounds with our algorithm was 11 % and precision was 4 %, which was of low performance compared to two monkey calls that were also targeted by the system. However, overall we had results comparable to other studies [19]. Please see Heinicke and colleagues [19] for further details about the algorithm and its performance metrics that were assessed in a separate study.

Alternatively, the Issa recordings were processed manually with the aid of the acoustic software TRITON [43], where long-term spectral averages (LTSA) were computed by the program on a daily basis, per SPATU, to produce a single 24 h LTSA. Researchers then scanned LTSAs to find areas with concentrated spectral energy within the frequency bandwidths in which chimpanzees usually call, i.e., below 6 kHz. Such areas were then zoomed into using 10 min windows, and listened to, in order to verify whether a chimpanzee call really was present in the recording. Hence, there was no automated analysis of the Issa data, nor any such validation study. It is important to note that data collection and processing had been independently done for both field sites before the present study was conceived. This is why chimpanzee drums were never documented in the Issa dataset while at Taï chimpanzee drumming is well known [34] and was the primary sound of interest.

In both PAM datasets we combined multiple chimpanzee detections into a single event when the time lag between the end and start of consecutive chimpanzee drums or calls was less than one minute. ARUs in Taï were intentionally spaced at a minimum distance of 1.2 km from each other to reduce the probability of detecting the same chimpanzee drum or pant hoot on multiple devices. However, this still occurred in some cases; therefore we removed multiple detections on neighboring ARUs of the same drum or call in the PAM data by comparing the time and date stamp of the call. Only the detection with the earliest time stamp was kept in the dataset based on the reasoning that whichever device recorded the chimpanzee call event first, the source individual must have been closest to this device. We did the same for Issa call events, where devices were spaced much closer together (minimum distance of 420 m) for the purposes of addressing additional research questions [36]. For each combination of recording day and device, we scored whether *at least one* chimpanzee drum or call event had occurred (1) or not (0), and this was done for both datasets to prepare them for comparable statistical analyses.

There is a high likelihood that we underestimated the true number of chimpanzee drums and pant hoots in both datasets due to the different methods of data processing, since obtaining the actual number could only be achieved if we had listened to and annotated all recordings which is not feasible for either dataset. For example, an individual listening to 8 hours of recordings per day would need 4 years (Taï data) or 10 years (Issa data) to manually listen to and annotate all the sounds. However, we are confident that even with a low number of detections we can still obtain a representative dataset of the spatiotemporal distribution of drums and pant hoots at each site since we have no reason to expect a bias for any particular device. To this end, we ensured that all ARUs or SPATUs had identical specifications and were deployed in the same manner at their respective field sites. Additionally, we found no bias for ARUs in detecting drum events during a validation study of the algorithm [19] developed for automated data analyses of the Taï recordings (permutation test of observed versus expected drum detections per ARU: *p* = 0.15).

#### Indices of chimpanzee activity

The Taï Chimpanzee Project (TCP) has been running for more than 35 years where neighboring chimpanzee communities have been habituated to the presence of researchers [30]. The ARU sampling grid overlapped two habituated chimpanzee communities, South and East groups, and at the time of the study the South group had been habituated since 1997 and the East group since 2007. Field assistants of the TCP regularly conducted all day focal follows of independent individuals (>5 years of age), collecting standardized behavioral data. The location of the focal individual was continuously recorded on a map with 500 m by 500 m grid cells overlaying the Taï research project area (~100 km^2^) to track ranging behavior and document the identity and number of accompanying chimpanzees in the focal’s party [30]. The map was projected onto a UTM coordinate system using ground-truthed GPS data to determine the center point of each grid cell. We then calculated chimpanzee activity in hours, for each grid cell of the Taï research area corresponding to the South and East groups’ territories, to obtain a direct measure of chimpanzee ranging and space-use. This was calculated by multiplying the size of a particular chimpanzee party by the number of minutes it was observed in a particular grid cell and dividing this by 60. Chimpanzee activity hours for each grid cell were then calculated on a daily basis for the 7 months corresponding to the same 7 months the ARUs were recording (November 2011 to May 2012). Here, 1,818 h of focal follow data were available for the South (884 h of observation) and East (934 h) group, respectively, across a total of 120 days. If no focal follow data was available for a particular ARU recording day due to interruption in the data collection by field assistants (occurred for 49/137 ARU recording days) then those days were excluded from the statistical analysis. Therefore, a total of 88 days remained for the analysis, where both focal follow data of chimpanzees was available and ARUs had recorded, across the 7 months study period. The chimpanzee activity hours within a detection radius of 500 m and 1 km of each ARU, was then totaled for each day, including only those grid cells where the detection radius overlapped with the center point of the grid cell.

The chimpanzees of the Issa valley were not habituated at the time of the study, and habituation had not yet started. However, surveys are regularly conducted in the region to permit monitoring of chimpanzee abundance [40]. Here, we used an indirect measure of chimpanzee activity in the area by counting the number of fresh nests (*N* = 101 fresh nest groups, group size range: 1- 26 nests) and encounters of other chimpanzee traces (*N* = 92: fresh feces (10), fresh feeding remains (1), vocalizations (32), and sightings of chimpanzees (49)) collected opportunistically while trying to find and locate chimpanzee nesting sites, or walking to and from line transects in the surrounding region. The data were therefore collected *ad libitum* throughout the Issa valley study area. The location of every encounter and fresh nest site found was recorded using a GPS in the field. Again, for a detection radius of 500 m and 1 km around each SPATU, the number of fresh nests and encounters located within the given detection radius for each recording device was summed, per day. Every unique encounter, regardless of type, was scored as a single indirect chimpanzee observation. This gave us the total number of indirect observations of chimpanzees within the respective radii of each SPATU found on a daily basis during the 11 months study period. All SPATU recording days were included in the analysis since it was possible for researchers to collect indirect observations every day during the study period.

At both sites, data were collected non-invasively. Research at Taï was approved by the Ethical Board of the Max Planck Society and was conducted with permissions from the Ministère de la Recherche Scientifique in Côte d’Ivoire, and adhered to the rules and regulations governing animal research in Germany and the EU. Data collection methods at Issa were approved by the Tanzania Wildlife Research Institute (TAWIRI) and adhered to the legal requirements of Tanzania and the American Society of Primatologists’ Principles for the Ethical Treatment of Non‐Human Primates.

### Statistical analyses

We conducted all statistical analyses using R version 3.1.3 [44], including the calculations of chimpanzee activity hours and indirect observations within the two detection radii for each recording device per day at their respective field sites. For the Taï dataset, we tested whether our direct measure of chimpanzee activity hours within the vicinity of the ARU (500 m and 1 km detection radius, respectively) predicted detections of chimpanzee drums on a daily basis at that device using Generalized Linear Mixed Models (GLMMs) [45], one for each detection radius. Similarly, we used GLMMs for the Issa dataset to test whether the number of indirect chimpanzee observations (the sum of fresh nests and encounters) within a 500 m and 1 km, respectively, detection radius of a SPATU predicted the number of chimpanzee calls detected by a device, again on a daily basis. All mixed models had a binomial error structure and logit link function [46] and the response was always whether or not a device had detected a chimpanzee sound event on a particular day (0/1). The final sample size for the Taï model was 1410 unique ARU-recording days, and for the Issa model 2470 unique SPATU-recording days. Models were run using the function ‘glmer’ of the R package ‘lme4’ [47]. GLMMs were fitted separately for the two field sites (see Additional file 1 for full model descriptions). At Taï, even though devices were set to record 6 h per day there was variation in recording effort due to technical difficulties (mean = 5.9 h, range = 0.5–6 h); therefore we included the log of the number of recording hours per day as an offset term into these models to control for this variation. Both test predictors, chimpanzee activity hours (Taï data) and number of indirect observations (Issa data), were z-transformed to a mean of zero and standard deviation of one before running the respective models [48]. Models included the identity of the recording device (ARU or SPATU) as a random effect as well as the random slopes for all fixed effects within the random effect of recording device [49, 50]. A full versus null model comparison was conducted first for every model using a likelihood ratio test with the R function ‘anova’ [46]. The null model comprised only the random effect, random slopes and in the case of the Taï models, also the offset term. For all models we verified model stability by ensuring model estimates did not vary strongly when recording devices were dropped one at a time. For the Taï dataset, we also had data on daily rainfall (mm). Since we expected rain to affect the ability of an ARU to properly record chimpanzee sounds, we fitted the same two Taï GLMMs (with a 500 m and 1 km detection radius, respectively) again but included rain as a control fixed effect after z transformation. However, rainfall data were at times missing which reduced the overall sample size further; therefore, we report the results for the model without rain as a control since this includes all the data collected and, most importantly, because rain did not affect ARU detection probability and did not change the results for Taï (see Additional file 2). For all GLMMs, the significance of individual predictors was assessed with a likelihood ratio test, again using the R function ‘anova’ [46], for which we report the Chi-square test statistic, as this is recommended to reduce the probability of making a type I error [50].

## Results

### Effect of chimpanzee ranging on ARU drum detections

**Fig. 1:**
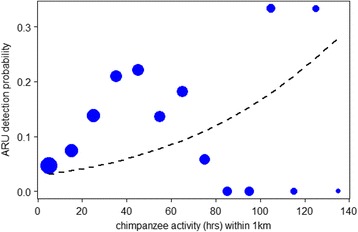
Chimpanzee drum detection probability at ARUs versus chimpanzee ranging activity within a 1 km radius of the device. Data were collected across 7 months in Taï forest where the probability that an ARU detected a chimpanzee drum was influenced by nearby chimpanzee ranging activity (est ± SE: 0.408 ± 0.14, X^2^ = 5.90, df = 1, *N* = 1410, *P* = 0.015). The dashed line shows the results of the fitted model per six hours of ARU recording effort. For plotting purposes only, data points were binned per 10 h of chimpanzee activity to obtain a mean detection probability per bin (*blue points*). The relative area of the circles corresponds to the log of the number of data points (range = 2 to 1111) per bin

There was a total of 233 chimpanzee events found in the Taï dataset using the algorithm (171 drums, 41 vocalizations, 21 drums with vocalizations) ranging from 0 to 47 drum events per ARU. Since the dataset was composed primarily of chimpanzee drums given that this was the sound targeted by the algorithm, we refer to these data collectively as drums hereafter for ease of distinction from the Issa data. We found that chimpanzee activity had a positive influence on the detection of chimpanzee drums on ARUs for the 88 days of data during the 7 months study period but only within a 1 km ARU detection radius (GLMM est ± SE = 0.408 ± 0.14, X^2^ = 5.90, df = 1, *N* = 1410, *P* = 0.015; Fig. 1). The effect was weaker for a 500 m ARU detection radius (0.287 ± 0.13, X^2^ = 3.50, df = 1, *N* = 1410, *P* = 0.061; see also Additional file 3) meaning that chimpanzee ranging activity correlated in space and time with ARU drum detections recorded within a 1 km radius, but only to a limited extent within a 500 m radius of an ARU.

**Fig. 2:**
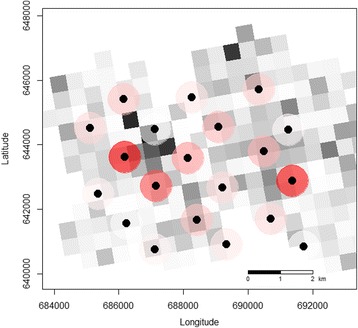
Chimpanzee ranging activity per 500 m by 500 m grid cell plotted alongside ARU drum detections. Ranging activity was the sum of the number of hours individual chimpanzees were observed per grid cell during focal follows across the 7 months study period. The darker the squares the higher the chimpanzee activity in that grid cell and the darker the red the greater the number of drum detections within a 500 m detection radius of the respective ARU (*black points*)

**Fig. 3:**
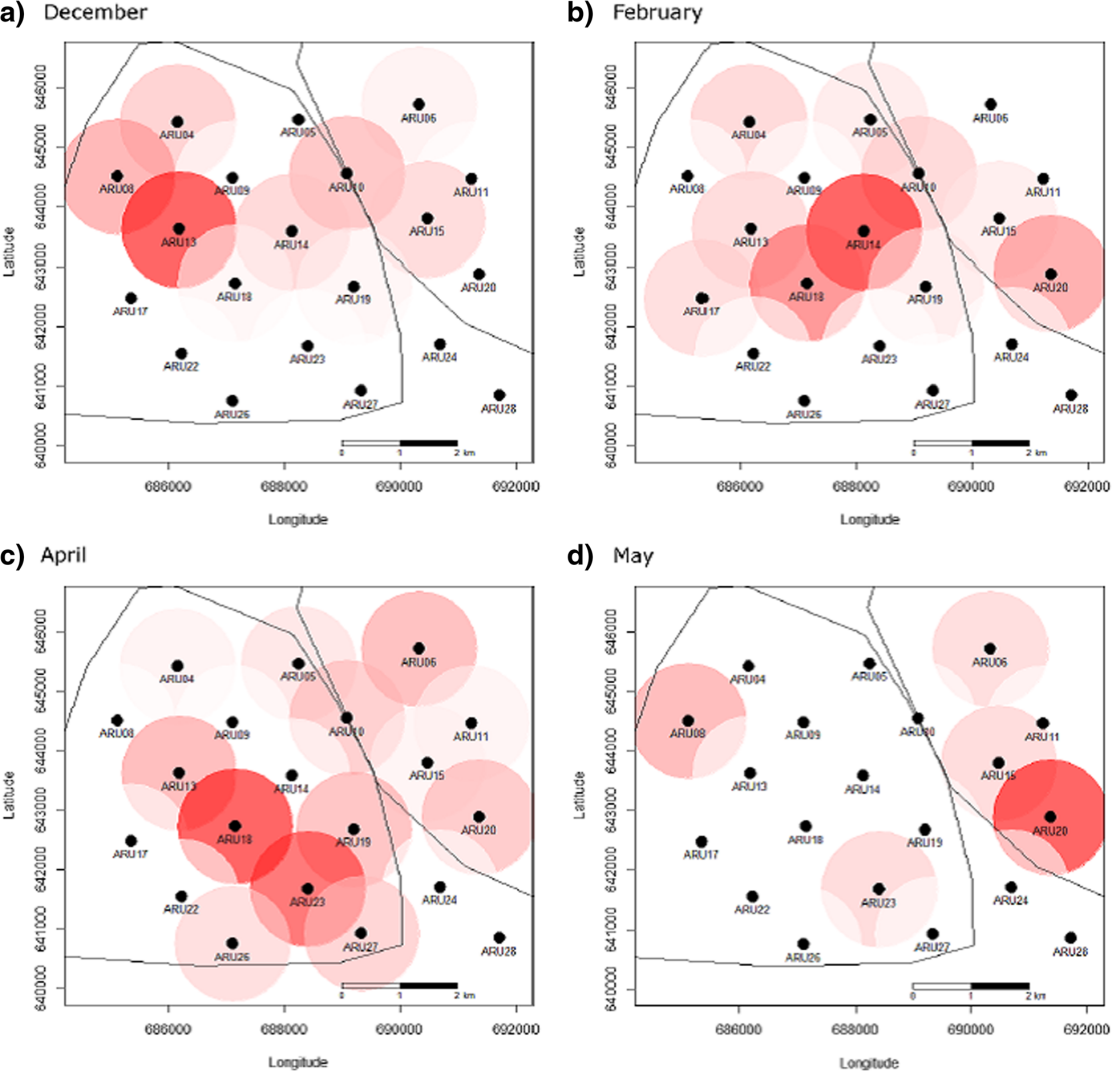
Changes in monthly centers of chimpanzee drumming activity illustrated by plotting PAM data. Drums detected on ARUs (*black points*) in Taï forest overlapped with two habituated chimpanzee communities. These 4 months (panels **a**-**d**) show the extent of the variation observed during the 7 months study period. The solid black lines outline the known home ranges for the two research communities (MCP 95 %: Kouakou et al. [6]). The detection distance of the ARU is shown at its maximum value of 1 km and the darker the color within the circle the greater the number of drums recorded on the respective device

Additionally, mapping all the ARU data (137 days of recordings) collected during the 7 months study period and comparing them to the well-known territory limits of the two habituated chimpanzee communities at Taï, revealed a pattern in the spatial distribution of drumming events detected on ARUs. Using already published home ranges of the two chimpanzee groups from long-term data (MCP 95 % [6]), we observed two locations with a high number of ARU drumming events in the west and east of the grid that corresponded roughly to the known whereabouts of the two habituated chimpanzee groups, the South and East group respectively (Fig. 2 and Fig. 3). However, the spatial distribution of ARUs was most comprehensive for the South group where the ARU grid covered almost the entire known home range of that habituated chimpanzee community, and here the greatest drumming activity on ARUs was found in the center (Fig. 2). With regards to the East group, the ARU grid only covered half of what was known as that chimpanzee community’s home range due to the loss of functioning ARUs during civil unrest in the country. Interestingly, when the PAM data were plotted on a monthly basis, drum detections were distributed across all ARUs and were not always highest in the core of the home range as predicted (Fig. 3).

### Effect of indirect chimpanzee activity on SPATU call detections

**Fig. 4:**
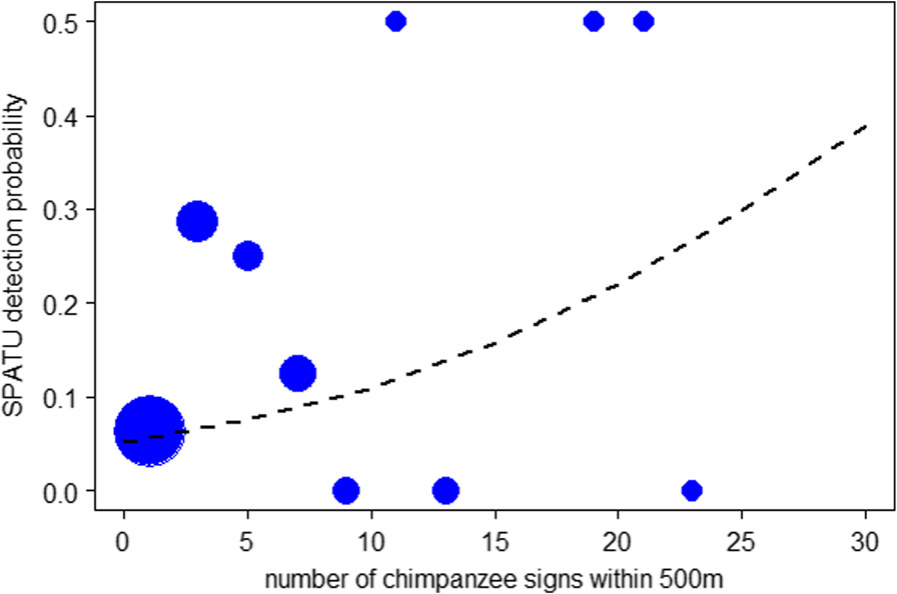
Chimpanzee call detection probability on SPATUs versus number of indirect observations (fresh nests and chimpanzees signs) nearby. At Issa, the probability of detecting a chimpanzee call on a SPATU was predicted by the total number of chimpanzee signs found within a 500 m radius (est ± SE: 0.126 ± 0.047, X^2^ = 4.06, df = 1, *P* = 0.044, *N* = 2470). The fitted model results are depicted with a dashed line. Again, for plotting purposes only, data points were binned for every two observations of indirect chimpanzee activity to obtain a mean detection probability per bin (*blue points*). The relative area of the circles corresponds to the log of the number of data points (range: 1 to 2427) per bin

**Fig. 5:**
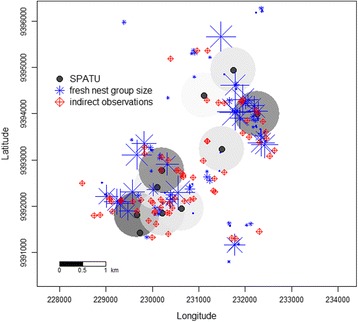
Spatial distribution of the 10 SPATUs located in Issa valley, Tanzania. The proportion of chimpanzee vocalizations detected on each SPATU (*black points*) for the 11 months study period, with respect to the locations of fresh chimpanzee nests (*flake*: area covered depicts the number of nests (1–26) in that location) and all other indirect chimpanzee observations (*diamonds with crosses*) are plotted. The darker the gray within the 500 m detection radius of the SPATU the greater the number of chimpanzee pant hoots recorded on the respective device

A total of 690 chimpanzee calls were in the Issa dataset, ranging from 0 to 159 call events per SPATU. In the Issa valley, where unhabituated chimpanzees roam across a savanna-woodland mosaic, we found a positive effect of the number of indirect chimpanzee observations within a radius of 500 m of a SPATU on its detection of chimpanzee calls (GLMM est ± SE: 0.13 ± 0.047, X^2^ = 4.06, df = 1, *N* = 2470, *P* = 0.044; Fig. 4 and Fig. 5) but not within a radius of 1 km (est ± SE: 0.184 ± 0.14, X^2^ = 2.15, df = 1, *N* = 2470, *P* = 0.15).

## Discussion

This study demonstrates that detections on autonomous recording devices, for two types of long-distance acoustic signals produced by chimpanzees, reflected direct and indirect indices of chimpanzee presence collected by field workers. Specifically, we found that the number of hours Taï chimpanzees were observed ranging in a given grid cell strongly predicted the probability of detecting a chimpanzee drum on an ARU located up to 1 km away (Fig. 1). At a second field site in the Issa valley, we also found that *ad libitum*, indirect observations of chimpanzees predicted detections of pant hoots and screams on SPATUs already within a 500 m radius (Fig. 4). These results are all the more promising since both datasets suffered from technical problems during data collection (typical for innovative solutions to field challenges) that resulted in discontinuous recording effort in their respective study periods. Additionally, the latter dataset came from unhabituated chimpanzees thereby further validating this method for monitoring poorly known and difficult to study populations.

The finding that the relationship was weaker within a smaller detection radius of 500 m for ARUs at Taï may be a consequence of many factors, particularly the propagation properties of a low frequency drum sound in a tropical rainforest. Chimpanzee drums are known to propagate well in a closed canopy habitat, up to 1 km, suggesting this sound is adapted for long distance communication [34, 35]. Also drum sounds were often confused with other background noise, namely thunder, rain, tree falls, and airplanes when using our algorithm [19] and as such many drums may have gone undetected. In fact, the algorithm used to process the Taï data had low overall performance, as was demonstrated in a previous study [19]. Hence, there are likely many more drums present in the data that were not extracted by our algorithm. For example, during all day focal follows of the South group chimpanzees we calculated an average rate of 2.47 pant hoots per hour for adult males (*N* = 5) and 0.64 pant hoots per hour for adult females (*N* = 4). The frequency of buttress drums was much lower overall, with only 0.9 drums per hour on average for adult males and 0.07 drums per hour, on average, for adult females. Therefore, we are undoubtedly missing many chimpanzee drum events in our Taï dataset due to the automated method of drum detections using our customized algorithm [19] and are likely to have missed some pant hoots in the Issa data as well due to human error [36]. These data processing methods, coupled with the fact that devices are fixed at a location, meant that chimpanzees had to pass within the detection radius to have even the possibility of their calls being recorded. Hence, a low sample size of sound events, particularly drums, may also contribute to the generally weak effects observed (Fig. 1 and Fig. 4). However, despite the low numbers of sound events in both datasets the PAM data still correlated with indirect and direct indices of chimpanzee ranging activity and were therefore still informative.

Alternative to chimpanzee drums, high frequency sounds, such as screams, which are also an integral component of the pant hoot [34], generally attenuate much quicker than low frequency sounds [51]. Therefore, chimpanzee calls at Issa may simply not propagate as far as 1 km which may be why indirect indices had no effect on call detections within a 1 km radius of a SPATU. However the Issa valley is characterized by a lot of open woodland-savanna which reduces the reverberation of sounds but the effects of shifting elevation and steep valleys would also be expected to disrupt call propagation [36, 51]. Due to these various conflicting effects more work needs to be done to investigate call transmission in such a habitat.

Interestingly, some of the highest rates of chimpanzee activity, direct and indirect, appeared to be associated with a lowered detection probability on ARUs and SPATUs at Taï and Issa, respectively (Figs. 1 and 4) although not consistently. This seems counterintuitive at first, but may in fact be a consequence of the long distance signals, pant hoots and drums, that were targeted in this study. The principal function of these long range sounds is for chimpanzees to maintain contact with other parties of their group [33, 37]. Therefore, although detection probability of these sounds is expected to increase when there are more chimpanzees, it may be that high values of chimpanzee activity hours reflect larger, cohesive parties, where individuals are in visual contact with multiple members of their community, reducing the need for frequent use of long distance calls or drums. Importantly, the relationship between long range sounds and chimpanzee presence might then become blurred at high rates of chimpanzee activity (e.g., see large values of chimpanzee activity in Figs. 1 and 4), and as such hamper monitoring efforts. This suggests that it may be fruitful to also incorporate short range calls, for example chimpanzee grunts, not just long distance sounds into any PAM scheme. Such a system would provide a more complete overall window into any given species’ vocal behaviour and thus, ranging patterns.

Identifying patterns in territory use is considered essential information for understanding ranging behaviour of many large mammals [52–54]. By visually tracking PAM data for just 7 months we were able to detect Taï chimpanzee drums produced by two neighboring chimpanzee communities, the habituated South and East groups. In addition, it appeared that both groups used different parts of their territories on a monthly basis (Fig. 3). However, while the South community had the greatest number of ARU drum detections in the core of their territory (Fig. 2), this was not true for the East group where most drumming events were detected on ARUs very close to the South group border (Fig. 2 and 3). This may have resulted from a high frequency of intergroup interactions, where chimpanzees often engage in vocal exchanges near community boundaries including buttress drumming, which can be used for territorial defense [29, 30]. However, the ARUs did not cover the entire East group territory so we cannot be sure, but it would suggest a pattern that is alternative to our original prediction, and should therefore be investigated further.

It is important to note that without individual identification of callers the determination of non-overlapping territories is only possible using PAM when there are several ARUs recording data per group. Devices should also be placed in a systematic grid design, as at Taï (Fig. 2), to avoid overlapping detection areas, as at Issa (Fig. 5). Still, drums along the border between chimpanzee communities would be difficult to assign to a single group when working with an unhabituated population, although PAM activity prior to arriving at the border could help to distinguish this if recordings are made at a high spatiotemporal resolution. It is recommended that the total detection area covered by the ARUs should cover at least the minimum home range size known for that species. For example, at Issa we cannot infer about the territory size or number of groups because a study area of only 12 km^2^ is much smaller than the smallest reported home range size for savanna-woodland chimpanzees (72 km^2^ [25]).

With increasingly sophisticated data processing techniques becoming available, automated individual identification of primate vocalizations should also become feasible in the future, permitting acoustic localization of individual callers, allowing us to map territories, individual movement patterns, and associations [14]. Generally, ARUs expand the spatial and temporal scale of data collection, also facilitating longitudinal data on multiple groups simultaneously. The application of PAM could therefore be extended to address questions regarding intergroup dynamics between neighboring groups which can rarely be done using traditional observational methods of single individuals from a single group, as well as how individuals living within fission-fusion species coordinate movement among sub-groups [55–57]. With the aid of radio transmission, PAM data can also be centralized and processed in near real-time if data mining methods continue to improve for the automated detection of sounds from continuous recordings [18, 19, 36, 58, 59].

## Conclusions

The present study has demonstrated the usefulness of remote acoustic sensing for accurately reflecting ranging patterns in three communities of wild apes. Our results have shown that PAM is a useful method to employ for the study of other, especially unhabituated, wild chimpanzee populations, to learn about group territories and their spatiotemporal patterns of habitat use. Additionally, PAM is non-invasive and causes negligible disturbance to wildlife. There is a strong caveat, however, accompanying our recommendation, which is that PAM relies on animals vocalizing; therefore, silent individuals will go undetected. Since wild primates may adapt their vocal behavior, especially by becoming quieter in areas with high poaching pressure [60], care should be taken when applying this method for the first time in such regions. That notwithstanding, PAM can be used to monitor presence, detection rates, and occupancy probabilities [32], as well as territory use and group ranging patterns (this study). Whereas we focused on a single species here, our findings can easily be adapted to other territorial mammals that use long-distance acoustic signals to communicate such as lions [61], spotted hyenas [62], and wolves [63].

With the advent of wireless technologies, we expect that PAM will become easier to implement, particularly over larger geographical scales; however, there are still many improvements needed before this method can be widely employed. In particular, PAM would benefit from further interdisciplinary research into automated sound recognition and classification methods since data analysis is still a limiting factor for continuous and large scale acoustic monitoring schemes. As automated approaches to data processing become available in real-time [18], wildlife managers will then be able to rapidly and efficiently collect, process, and incorporate empirical data into conservation policy. Tracking spatiotemporal shifts in animal activity using remote acoustic data could then be used to inform conservation priorities, identify key resources, as well as threats (e.g., gunshots and logging). To date, we have only begun to explore the power of PAM for addressing research questions in conservation science and behavioral ecology and we encourage researchers of other taxa to add this promising new method to their field work toolbox.

## Abbreviations

ARU, autonomous recording unit; PAM, passive acoustic monitoring; SPATU, solar powered acoustic transmission unit; TCP, Taï Chimpanzee Project

## Additional files

Additional file 1: Description of the GLMMs fitted for PAM data collected from both field sites. (DOCX 15 kb) Additional file 2: GLMM results for the effect of chimpanzee activity on ARU drum detections of chimpanzees at Taï, with daily rainfall (mm) added as a control fixed effect. We checked for collinearity using variance inflation factors derived using the function ‘vif’ of the package ‘car’ [1] applied to a standard linear model lacking the random effects. The effect of chimpanzee ranging activity remained significant at a one kilometer detection radius, and rain had no effect on ARU detection probability (*N* = 1391). Similarly, results remained a trend (*P* = 0.065) with a 500 m detection radius (see Additional file 3 and main manuscript). (DOCX 15 kb) Additional file 3: Chimpanzee drum detection probability at ARUs and chimpanzee ranging activity within a 500 m detection radius of the device in Taï (est ± SE: 0.287 ± 0.12, X^2^ = 3.50, df = 1, *P* = 0.061, *N* = 1410). The dashed line shows the results of the fitted model per 6 hours of ARU recording effort. Data points were binned per 10 hours of chimpanzee activity to obtain a mean detection probability per bin (blue circles). The relative area of the circles corresponds to the log of the number of data points (range: 1 to 1291) per bin. (DOCX 662 kb)
