# Supplementary material for: Passive acoustic monitoring reveals group ranging and territory use: a case study of wild chimpanzees (Pan troglodytes)
Source: Front Zool. 2016 Aug 8;13:34. doi: 10.1186/s12983-016-0167-8 (PMC4977853; doi:10.1186/s12983-016-0167-8)
Supplement: Additional file 1: — Description of the GLMMs fitted for PAM data collected from both field sites. (DOCX 15 kb) [file 12983_2016_167_MOESM1_ESM.docx]

Passive acoustic monitoring reveals group ranging and territory use: a case study of wild chimpanzees (*Pan troglodytes*)

Ammie K. Kalan^1^, Alex K. Piel^2, 3^, Roger Mundry^1,4^, Roman M. Wittig^1, 5^, Christophe Boesch^1,6^, Hjalmar Kühl^1, 7^

^1^ Department of Primatology, Max Planck Institute for Evolutionary Anthropology, Deutscher Platz 6, 04103 Leipzig, Germany

^2^School of Natural Sciences and Psychology, Liverpool John Moores University, James Parsons Building, Rm 653, Byrom Street, Liverpool L3 3AF UK

^3^Ugalla Primate Project, Kigoma, Tanzania

^4^ Department of Developmental and Comparative Psychology, Max Planck Institute for Evolutionary Anthropology, Deutscher Platz 6, 04103 Leipzig, Germany

^5^Taï Chimpanzee Project, Centre Suisse de Recherches Scientifiques, BP 1301, Abidjan 1, CI

^6^Wild Chimpanzee Foundation, Deutscher Platz 6, 04103 Leipzig, Germany

^7^German Centre for Integrative Biodiversity Research (iDiv) Halle-Jena-Leipzig, Deutscher Platz 5e, 04103 Leipzig, Germany

**Additional File 1**

Description of the GLMMs fitted for PAM data collected from both field sites.

|  | Response | Fixed effects | Random effects | Random slopes | Offset |
| --- | --- | --- | --- | --- | --- |
| Taï, Côte d’Ivoire (N=1410) | ARU drum detections per day & device (0/1) | Chimp activity hours (500m and 1km radius) | ARU (20) | For ARU within chimp activity | Log(recording hours per day & device) |
| Issa valley, Tanzania (N=2470) | SPATU call detections per day & device (0/1) | Number of indirect chimp observations (500m and 1km radius) | SPATU (10) | For SPATU within # of indirect observations | None needed |

Identical but separate models were fitted for the two detection radii of 500m and 1km for each site.
