# Supplementary material for: Passive acoustic monitoring reveals group ranging and territory use: a case study of wild chimpanzees (Pan troglodytes)
Source: Front Zool. 2016 Aug 8;13:34. doi: 10.1186/s12983-016-0167-8 (PMC4977853; doi:10.1186/s12983-016-0167-8)
Supplement: Additional file 2: — GLMM results for the effect of chimpanzee activity on ARU drum detections of chimpanzees at Taï, with daily rainfall (mm) added as a control fixed effect. We checked for collinearity using variance inflation factors derived using the function ‘vif’ of the package ‘car’ [1] applied to a standard linear model lacking the random effects. The effect of chimpanzee ranging activity remained significant at a one kilometer detection radius, and rain had no effect on ARU detection probability (N = 1391). Similarly, results remained a trend (P = 0.065) with a 500 m detection radius (see Additional file 3 and main manuscript). (DOCX 15 kb) [file 12983_2016_167_MOESM2_ESM.docx]

Passive acoustic monitoring reveals group ranging and territory use: a case study of wild chimpanzees (*Pan troglodytes*)

Ammie K. Kalan^1^, Alex K. Piel^2, 3^, Roger Mundry^1,4^, Roman M. Wittig^1, 5^, Christophe Boesch^1,6^, Hjalmar Kühl^1, 7^

^1^ Department of Primatology, Max Planck Institute for Evolutionary Anthropology, Deutscher Platz 6, 04103 Leipzig, Germany

^2^School of Natural Sciences and Psychology, Liverpool John Moores University, James Parsons Building, Rm 653, Byrom Street, Liverpool L3 3AF UK

^3^Ugalla Primate Project, Kigoma, Tanzania

^4^ Department of Developmental and Comparative Psychology, Max Planck Institute for Evolutionary Anthropology, Deutscher Platz 6, 04103 Leipzig, Germany

^5^Taï Chimpanzee Project, Centre Suisse de Recherches Scientifiques, BP 1301, Abidjan 1, CI

^6^Wild Chimpanzee Foundation, Deutscher Platz 6, 04103 Leipzig, Germany

^7^German Centre for Integrative Biodiversity Research (iDiv) Halle-Jena-Leipzig, Deutscher Platz 5e, 04103 Leipzig, Germany

**Additional File 2**

GLMM results for the effect of chimpanzee activity on ARU drum detections of chimpanzees at Taï, with daily rainfall (mm) added as a control fixed effect. We checked for collinearity using variance inflation factors derived using the function ‘vif’ of the package ‘car’ [1] applied to a standard linear model lacking the random effects. The effect of chimpanzee ranging activity remained significant at a one kilometer detection radius, and rain had no effect on ARU detection probability (N= 1391). Similarly, results remained a trend (P=0.065) with a 500m detection radius (see Additional file 3 and main manuscript).

|  | Estimate | SE | X^2^ | df | P value |
| --- | --- | --- | --- | --- | --- |
| chimpanzee activity (hrs) | 0.403 | 0.14 | 5.74 | 1 | 0.017 |
| rainfall | -0.026 | 0.11 | 0.083 | 1 | 0.82 |
