# Supplementary material for: Passive acoustic monitoring reveals group ranging and territory use: a case study of wild chimpanzees (Pan troglodytes)
Source: Front Zool. 2016 Aug 8;13:34. doi: 10.1186/s12983-016-0167-8 (PMC4977853; doi:10.1186/s12983-016-0167-8)
Supplement: Additional file 3: — Chimpanzee drum detection probability at ARUs and chimpanzee ranging activity within a 500 m detection radius of the device in Taï (est ± SE: 0.287 ± 0.12, X2 = 3.50, df = 1, P = 0.061, N = 1410). The dashed line shows the results of the fitted model per 6 hours of ARU recording effort. Data points were binned per 10 hours of chimpanzee activity to obtain a mean detection probability per bin (blue circles). The relative area of the circles corresponds to the log of the number of data points (range: 1 to 1291) per bin. (DOCX 662 kb) [file 12983_2016_167_MOESM3_ESM.docx]

Passive acoustic monitoring reveals group ranging and territory use: a case study of wild chimpanzees (*Pan troglodytes*)

Ammie K. Kalan^1^, Alex K. Piel^2, 3^, Roger Mundry^1,4^, Roman M. Wittig^1, 5^, Christophe Boesch^1,6^, Hjalmar Kühl^1, 7^

^1^ Department of Primatology, Max Planck Institute for Evolutionary Anthropology, Deutscher Platz 6, 04103 Leipzig, Germany

^2^School of Natural Sciences and Psychology, Liverpool John Moores University, James Parsons Building, Rm 653, Byrom Street, Liverpool L3 3AF UK

^3^Ugalla Primate Project, Kigoma, Tanzania

^4^ Department of Developmental and Comparative Psychology, Max Planck Institute for Evolutionary Anthropology, Deutscher Platz 6, 04103 Leipzig, Germany

^5^Taï Chimpanzee Project, Centre Suisse de Recherches Scientifiques, BP 1301, Abidjan 1, CI

^6^Wild Chimpanzee Foundation, Deutscher Platz 6, 04103 Leipzig, Germany

^7^German Centre for Integrative Biodiversity Research (iDiv) Halle-Jena-Leipzig, Deutscher Platz 5e, 04103 Leipzig, Germany

**Additional File 3**


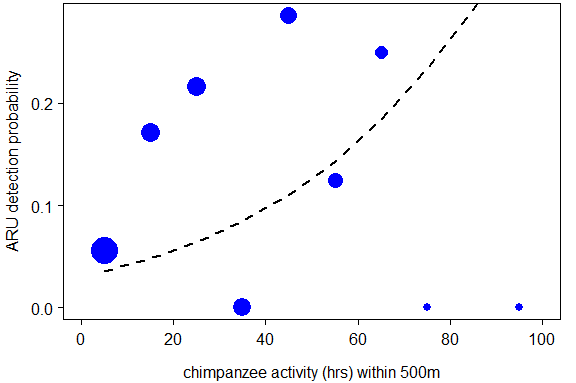


Chimpanzee drum detection probability at ARUs and chimpanzee ranging activity within a 500m detection radius of the device in Taï (est ± SE: 0.287 ± 0.12, X^2^= 3.50, df= 1, P= 0.061, N= 1410). The dashed line shows the results of the fitted model per 6 hours of ARU recording effort. Data points were binned per 10 hours of chimpanzee activity to obtain a mean detection probability per bin (blue circles). The relative area of the circles corresponds to the log of the number of data points (range: 1 to 1291) per bin.
